# Identification of a novel QTL contributing to rust resistance in *Eucalyptus*

**DOI:** 10.1186/1753-6561-5-S7-P32

**Published:** 2011-09-13

**Authors:** Bruno M Lima, Juliana EC Teixeira, Rodrigo Gazaffi, Antonio AF Garcia, Dario Grattapaglia, Raphaelle KD Valle, Luis EA Camargo

**Affiliations:** 1 Departamento de Genética, Universidade de São Paulo - ESALQ/USP, Piracicaba, SP, Brazil; 2 Fibria, Jacareí, SP, Brazil; 3 EMBRAPA Recursos Genéticos e Biotecnologia, Brazilia, DF, Brazil; 4 Departamento de Fitopatologia e Nematologia, Universidade de São Paulo - ESALQ/USP, Piracicaba, SP, Brazil

## Background

The genus *Eucalyptus* has many species that are well adapted to a wide range of environmental conditions in Brazil. However, in some areas diseases are a limiting factor, among which the Eucalyptus rust caused by *Puccinia psidii* Winter stands out as a destructive pathogen of the Myrtacea. The growth of plants with high levels of infection is severely compromised and these plants end up being dominated by adjacent plants which ultimately leads to their death. The most efficient method to control the disease is through the use of resistant genotypes. A major effect rust resistance QTL has already been identified [1,2], however a residual variance stays unexplained. In this context, our study aimed to identify genomic regions containing quantitative resistance loci to rust based on segregation data of a S_1_ progeny from an inter-specific (*Eucalyptus grandis* x *E. urophylla*) resistant clone.

## Material and methods

The progeny used in this study was generated by the breeding program of Fibria and is composed of 90 S_1_ trees from the self-pollination of an *E. grandis* x *E. urophylla* hybrid tree. This hybrid (VCP-R) is heterozygous for a rust resistance locus [3]and highly resistant to rust. The plants were evaluated at two different sites with high incidence of rust starting in November 2005. Four disease severity evaluations were made at 90, 120, 150 and 180 days after planting by scoring the levels of disease using a four-score severity scale. Scores were used to calculate the area under the disease progress curve (AUDPC) for each plant and for BLUPs estimation. Genomic DNA was CTAB extracted from health young leaves and used in PCR amplifications of 121 microsatellite, 20 TRAP and 38 AFLP markers (totalizing 179 markers). The Onemap software [4]was used in linkage analysis. The estimated BLUPs were used in QTL mapping [5]. The QTLs were mapped by interval mapping (IM) and composite interval mapping (CIM).

## Results

**Figure 1:**
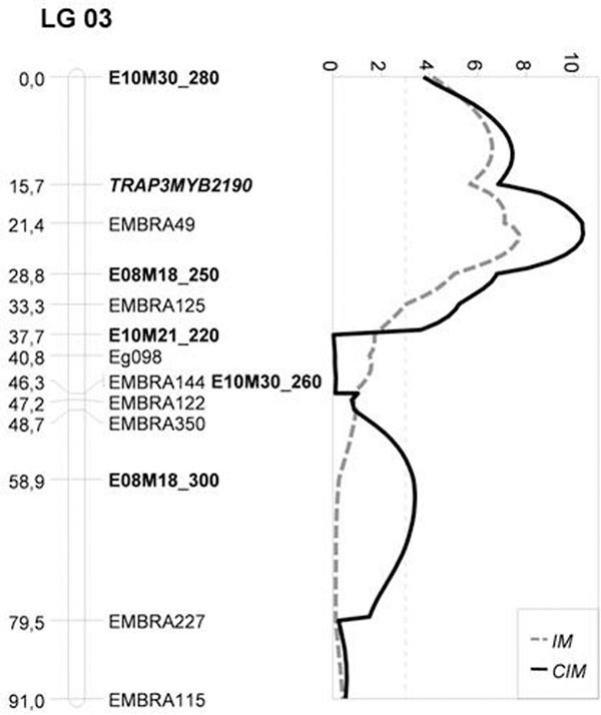
IM and CIM QTL mapping for Eucalyptus rust on linkage group 3.

Linkage analysis resulted in 11 linkage groups with 160 linked markers. The identity of the groups were as in [6]based on common microsatellite loci as references. The length of the map was 1075 cM with an average distance of 6.7 cM. IM analysis identified one QTL in linkage group 3 (LOD=7.7) with a large phenotypic effect that accounted for 28.5% of the variation (fig. 1). The negative additive effect was significant (p = 0.05), without dominance effects. QTL was located 1.6 cM from SSR EMBRA049 and 5.8 cM from AFLP E08M18_250. The CIM analysis mapped two QTLs, both located in linkage group 3. The peak LOD score (LOD=10.3) of QTL_1_, which explained 39.5% of the phenotypic variation, mapped in the same interval of the QTL identified in the IM analysis. It was located close to Embra049 (1.6 cM) and presented significant negative additive effects and no significant dominance effect. The second QTL (QTL_2_), with LOD=3.4, had a relative lower phenotypic effect (6,9%), mapped 3.1 cM close to the AFLP marker E08M18_300 AFLP. However, differently than QTL_1_, QTL_2_ presented both additive and dominance effects.

## Conclusions

The use of the AUDPC permitted to transform discrete variables (symptoms scores) into a continuous variable, which gives greater power in QTL detection. The effect of the reduced size of the S_1_ progeny in QTL detection was compensated by the BLUPs analysis, which considers only the genetic variation in QTL localization. QTL mapping was done by two different approaches (IM and CIM) that presented contrasting results. The non detection of QTL_2_ in the IM analysis was probably due to the limitations of the method (e.g., non-independence in different intervals when there are more than one QTL and low precision of the position by the presence of linked QTLs). Previous studies on the inheritance of resistance to rust in eucalyptus identified a locus with large phenotypic effect, but also pointed to the existence of minor effects [1,2]. Our results confirm the hypothesis of the presence of at least one minor effect locus, by data analysis in quantitative approach and CIM, as never used before in Eucalyptus rust resistance mapping. This work revealed the existence of a second rust resistance locus in addition to one with large effect locus previously mapped in linkage group 3. The identification of markers linked to resistance loci gives perspectives of MAS of resistant genotypes, which is desirable in the case of this disease due to the technical and economical difficulties of selecting resistant genotypes based solely on phenotypes since this biotrophic requires large areas and skilled labor. In addition this study opens possibilities for the positional cloning of the resistance genes together with the availability of the genome sequence of eucalyptus.

**Financial support.**Fibria, CNPq, CAPES, ESALQ-USP.
